# Comparative Effectiveness of Clostridial Collagenase Ointment to Medicinal Honey for Treatment of Pressure Ulcers

**DOI:** 10.1089/wound.2016.0720

**Published:** 2017-04-01

**Authors:** Adrienne M. Gilligan, Curtis R. Waycaster, Richard Bizier, Bong-Chul Chu, Marissa J. Carter, Caroline E. Fife

**Affiliations:** ^1^ Truven Health Analytics, Houston, Texas.; ^2^ Smith & Nephew, Fort Worth, Texas.; ^3^ Truven Health Analytics, Cambridge, Massachusetts.; ^4^ Truven Health Analytics, Santa Barbara, California.; ^5^ Strategic Solutions Inc., Cody, Wyoming.; ^6^ Intellicure, Inc, The Woodlands, Texas.

**Keywords:** pressure ulcer, debridement, granulation, epithelialization, clostridial collagenase ointment, medicinal honey

## Abstract

**Objective:** Compare enzymatic debridement using clostridial collagenase ointment (CCO) with autolytic debridement using medicinal honey in the hospital outpatient setting for treating pressure ulcers (PUs).

**Approach:** Retrospective deidentified electronic health records from 2007–2013 were extracted from the U.S. Wound Registry. Propensity score matching followed by multivariable analyses was used to adjust for selection bias and assess treatment effects comparing CCO-treated versus honey-treated PUs. Key outcomes included 100% granulation and epithelialization at 1 year.

**Results:** Five hundred seventeen CCO-treated PUs (446 patients) were matched to corresponding honey-treated PUs (341 patients). The majority of PUs were stage III (CCO 56%, honey 55%). CCO users had significantly fewer total visits (9.1 vs. 12.6; *p* < 0.001), fewer total selective sharp debridements (2.7 vs. 4.4; *p* < 0.001), and fewer PUs receiving negative pressure wound therapy (29% vs. 38%; *p* = 0.002) compared with honey.

**Innovation:** CCO-treated PUs were 38% more likely to achieve 100% granulation compared to honey-treated PUs at 1 year, *p* = 0.018. Mean days to 100% granulation were significantly lower for CCO-treated PUs (255 vs. 282 days, *p* < 0.001). CCO-treated PUs were 47% (*p* = 0.024) more likely to epithelialize at 1 year compared to PUs treated with honey. Mean days to epithelialization were significantly lower for PUs treated with CCO at 1 year (288 vs. 308 days; *p* = 0.011).

**Conclusion:** All stages of PUs treated with CCO achieved faster rates of granulation and subsequent epithelialization compared to PUs treated with medicinal honey as measured by real-world data collected in the hospital outpatient department care setting.

**Figure:**
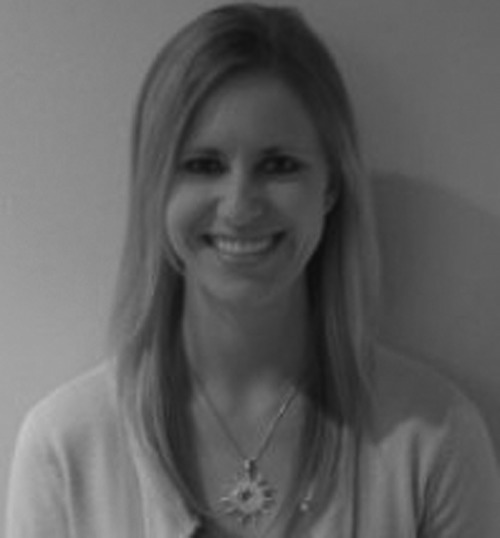
**Adrienne M. Gilligan, PhD**

## Introduction

Pressure ulcers (PUs) pose substantial clinical and economic challenges to patients, their families, and healthcare providers, annually affecting an estimated 2.5 million patients in the United States (US) at a substantial cost, estimated to range $9.1–$11.6 billion annually.^1–3^ Seen most commonly in seriously ill patients, the elderly, and infants due to underlying conditions impairing movement, PUs are an indicator of poor overall health status that may contribute to mortality risk.^4,5^ The prevalence of these high cost adverse events varies considerably by clinical setting, ranging from 0.4% to 38% in acute care hospitals, 2.2% to 23.9% in long-term care facilities, and 0% to 17% in home healthcare settings.^6–8^

Management of PUs is often complicated by the patient's underlying disease and associated pain, infection, and interference with functional recovery leading to lengthened hospital stays and increased costs of care.^8,9^ PU severity is categorized using a four stage classification system, ranging from stage I (intact skin, nonblanchable erythema) to stage IV (full thickness tissue loss exposing bone, tendon, or muscle).^10,11^ Management requires a multimodal care strategy, including nutritional assessment, body positioning, mobilization, and environmental factors (bedding, chairs, medical devices, *etc.*), as well as direct treatments to the wound itself.^11^ Treatment of chronic wounds such as PUs entails a systematic approach to preparing the wound bed for healing, consisting of debriding unhealthy and nonviable tissue, controlling exudate and edema, controlling infection and bacterial bioburden, maintaining moisture balance, and promoting healthy granulation tissue.^12–17^

Debridement, the removal of nonviable and poorly healing tissue, foreign material, and bacteria (biofilm) from a wound, is critical to preparing the wound bed for healing and facilitating wound epithelialization.^18–21^ Debridement methods have been described as surgical or sharp, mechanical, chemical, biological, autolytic, and enzymatic.^16,22^ These methods may be used singly or combined serially to optimize the debridement process. Some are considered “selective,” acting only on necrotic tissue, or “nonselective” that remove normal, as well as necrotic, tissue.^23^ The choice of debridement method depends on patient factors, wound type, likelihood of infection, available medical expertise, and care setting resources.^9,24^

Enzymatic wound debridement with clostridial collagenase ointment (CCO) has been used since the mid-1960s and is the only FDA approved enzymatic agent indicated for debriding chronic dermal ulcers and severely burned areas.^25^ Collagenase, a proteolytic enzyme derived from Clostridium histolyticum, breaks down collagen in necrotic tissue, selectively removing detritus without harming healthy tissue, facilitating granulation tissue formation and epithelialization.^22,26–31^ Other clinical advantages of enzymatic debridement with CCO include ease of application, minimal blood loss, and enhanced tissue proliferation.^32^

Autolytic debridement with support from topically applied medicinal honey is thought to work with the body's natural healing process to cleanse debris and necrotic tissue from the wound. Its activity has been attributed to its acidity increasing oxygen release from hemoglobin, decreasing the activity of destructive proteases, and osmotically drawing fluid from the wound bed to create an outflow of lymph. While there is some evidence supporting the use of honey in wound care, there is however a lack of comparative effectiveness data.^33,34^

## Clinical Problem Addressed

There is a lack of current evidence comparing enzymatic debridement with medicinal honey-based autolytic debridement as clinically effective therapies for the management of chronic wounds. The objective of this study was to compare clinical outcomes, particularly granulation and epithelialization, between enzymatic debridement with CCO and autolytic debridement with support from medicinal honey for the treatment and management of PUs in the hospital outpatient environment. Real-world observational data were used to enable more easily and quickly recruiting a larger patient population that is representative of actual clinical practices to support a better understanding of PU treatment beyond the controlled environment of a clinical trial.

## Materials and Methods

### Study design

This study of PU patients used a longitudinal observational case–control confirmatory design approach to compare matched treatment cohorts receiving CCO (cases) with controls treated with medicinal honey, analyzing the differences and risk factors in achievement of wound granulation and epithelialization over time.

### Data source

Data were drawn from the U.S. Wound Registry (USWR) database, comprising electronic health records (EHRs) from over 100 hospital-based outpatient wound centers in the US and Puerto Rico. The USWR is certified to meet the Health Information Technology for Economic and Clinical Health Act (HITECH Act) standards, including adherence to wound care quality measures developed by the USWR as a Qualified Clinical Data Registry (QCDR) and nationally recognized by the Centers for Medicare and Medicaid Services as part of the Physician Quality Report System.^35,36^ Clinical data are collected at the point of care using standardized codes and vocabularies with limited use of free text.^37^ All wound and ulcer types are included, such as diabetic foot ulcers, venous stasis ulcers, PUs, arterial ulcers, surgical wounds, traumatic wounds, vasculitic ulcers, arterial ulcers, sickle cell ulcers, inflammatory ulcers (*e.g.,* pyoderma gangrenosum), and ulcers related to skin disorders such as scleroderma. Ulcers are risk stratified for outcomes reporting using the Wound Healing Index. Interventions include dressings, compression bandaging, off-loading, cellular- and/or tissue-based therapies, hyperbaric oxygen therapy, negative pressure wound therapy, debridement, and antibiotics. Outcomes measured include healing or wound closure, surgical closure, death, and amputations.

The USWR EHR database was certified to satisfy the conditions set forth in Sections 164.514 (a)-(b)1ii of the Health Insurance Portability and Accountability Act (HIPAA) Privacy Rule regarding the determination and documentation of statistically deidentified data. Woodlands Institutional Review Board, acting as USWR's independent Institutional Review Board, has determined that retrospective analyses of deidentified HIPAA-compliant data described herein are exempt from the requirement of patient consent.

### Subject selection

Patients were selected who had at least one encounter record with a PU diagnosis code (International Classification of Diseases, Ninth Revision, Clinical Modification [ICD-9-CM] diagnosis codes 707.00–707.07, 707.09, and 707.20–707.25) in any diagnosis position between January 1, 2007, and December 31, 2012. The date of the first found PU diagnosis code during this period was the patient's index date. Patients were required to have been treated with either CCO or honey and have at least one more encounter with a PU diagnosis after the index PU event. Subjects were excluded who were treated with both CCO and honey during their follow-up, were younger than 18 years old at index, or whose PUs healed within 2 weeks postindex.

Patients and their wounds were followed from their index date until the earliest of 100% granulation tissue formation, epithelialization, or the end of the study period (December 31, 2012).

### Outcome measures

The primary outcome of interest was complete granulation tissue formation for 100% of the wound bed, measured as the percentage of the wound bed with granulation tissue formed. Achievement of 100% granulation (yes/no) and the time to the first recorded encounter noting 100% granulation were measured. If insufficient data were available for granulation, the percentage of necrotic tissue in the wound was assessed, representing the remaining part of the wound not yet granulated.

The secondary clinical end point of epithelialization was evidence of achieving complete epithelialization (yes/no) and time to complete epithelialization. Wounds were considered epithelialized when all of the following criteria were met: no wound exudate, wound area (length × width) <0.2 square centimeters, and tissue depth <0.1 cm or described as “partial thickness,” the wound overall was described as controlled or improved and not worsening, and the wound bed was characterized as epithelialized.

### Explanatory variables

The primary explanatory variable was PU treatment with either CCO or honey. Patients were grouped into mutually exclusive comparison cohorts based on receiving PU treatment with either CCO or honey. The following treatment parameters were calculated using the date of the initial wound care visit and each subsequent wound care visit for PU treatment: number of PUs treated, time to first application (days from index date to the first visit when treatment was applied), days of use (last date of application minus the first date of application), number of treatment episodes (defined by gaps between applications of at least 60 days), visits with treatment (number of visits when treatment was applied), application rate (days of use divided by application count), and percentage of treated visits (application count divided by total visit count).

The following PU characteristics were captured: arrival score for initial visit (ambulatory, cane/crutches, walker, wheelchair/scooter, and stretcher/bed), number of concurrent wounds, wound age at first visit, wound surface area at first visit, wound depth at first visit, PU stage (unstageable, II, III, or IV), PU location, wound exudate level (none, minimal, moderate, or heavy), periwound characteristics (normal, erythematous, macerated, and other), infection surrogate score (“green event” count over treatment time of PU, where “green events” occur when there is an antibiotic order, a laboratory test for bacterial culture, provider's EHR note of infection or oozing green pus from the wound), total visit count, visit frequency (visit count divided by days from first to last visit), total number of selective sharp debridements (visits with Current Procedural Terminology [CPT] codes 97597, 97598, or 11040–11047), debridement rate (debridement count divided by days from first to last visit), and concurrent therapies, including hyperbaric oxygen, negative pressure wound therapy, becaplermin gel, and cellular tissue-derived products (OASIS, Apligraf, Dermagraft, Integra, and PriMatrix).

Other explanatory variables included the following patient demographics and clinical characteristics: age, sex, payer, ethnicity, smoker, receiving palliative care, pain medication, antibiotics, antirejection drugs (immunocompromised), paralyzed, home healthcare, comorbidities (heart failure, coronary heart disease, end-stage renal disease [ESRD], peripheral vascular disease, hypertension, and diabetes).

### Analysis

Wounds receiving PU treatment with CCO were matched 1:1 to PUs treated with honey using the following propensity score–based matching approach. The goal of propensity score matching is to reduce bias by statistically emulating the comparator balance inherent in randomized clinical trials (RCTs) so that the distribution of observed baseline demographic and clinical characteristics is similar between treatment groups.^38^ Propensity scores for each wound were modeled using logistic regression with a binary indicator for membership in the CCO or honey PU cohorts as the dependent variable and a vector of the following independent variables at index or first PU visit: patient age, wound age, wound surface area, arrival score, number of concurrent wounds, infection surrogate score, ESRD (yes/no), PU stage, and patient paralyzed (yes/no). CCO-treated PUs (cases) were matched to honey-treated control PUs using the nearest neighbor matching technique, enforcing a caliper of 0.25 times the standard deviation (SD) of the propensity score to maximize the number of available controls (honey-treated PUs) for each CCO-treated PU.^39^ The balance achieved by propensity score matching was assessed using standardized differences comparing the pre- and post-match distributions of the independent variables included in the propensity score model.^38^

Dependent and independent variables were summarized descriptively, with categorical variables presented as the count and percentage of patients and continuous variables providing the number of observations, the mean, SD, and median. Statistical tests of significance for observed differences between treatment groups were conducted using Chi-square tests for categorical variables and t-tests for continuous variables.

Multivariate models were estimated postmatching to control for any remaining imbalances between cases and controls. Cox proportional hazard models were estimated to identify risk factors for the time from index date to granulation or epithelialization. The assumption of proportional hazards was tested plotting Kaplan–Meier observed survival curves and comparing them with Cox predicted curves from the same variable. Logistic regression models were estimated to examine the impact of patients' demographic, clinical, and treatment characteristics on granulation and epithelialization within a fixed time period (*e.g.,* 1-year follow-up). All models controlled for patients' demographic and clinical characteristics at baseline to estimate hazard and odds ratios (HR and OR) of each factor on granulation and epithelialization.

The threshold of statistical significance for all analyses was set a priori at 0.05. Statistical analyses were conducted using STATA SE, version 13 (STATA Corp, College Station, TX). Data management and analytic file building were conducted using SAS software, version 9.4 (SAS Institute, Inc., Cary, NC).

## Results

The final matched PU treatment cohorts meeting all selection criteria each included 517 wounds from 446 CCO-treated patients and 341 honey-treated patients found in the USWR from 2007 through 2012 (Table 1).

**Table 1.** Patient and wound sample attrition applying selection criteria

	*Patients*		*Wounds*	
*Selection Criteria*	n	*%*	n	*%*
PU diagnosis^a^ found January 1, 2007—December 31, 2012^b^ and ≥1 encounter in the USWR after the index date	9,202	100.0	20,330	100.0
At least 1 application of CCO or medicinal honey	2,711	29.5	7,823	38.5
At least 18 years old on the index date	2,685	29.2	7,752	38.1
Not treated with both CCO and medicinal honey	2,639	28.7	4,512	22.2
Healed within 2 weeks or had only one visit record in USWR	2,639	28.7	4,512	22.2
CCO cohort before matching	2,297	25.0	3,993	19.6
Medicinal honey cohort before matching	342	3.7	519	2.6
CCO cohort after matching	446	4.8	517	2.5
Medicinal honey cohort after matching	341	3.7	517	2.5

^a^ PU diagnosis required one of these ICD-9-CM diagnosis codes: 707.00–707.07, 707.09, or 707.20–707.25.

^b^ The first found PU diagnosis is the patient's index date.

CCO, clostridial collagenase ointment; PU, pressure ulcer; USWR, U.S. Wound Registry.

Patients were on average aged 64 to 66 years with the majority older than 65 years (CCO 59.0%, honey 52.8%; Table 2). Around two-thirds of patients were ambulatory on arrival (CCO 66.4%, honey 63.6%), with over half receiving home healthcare (CCO 59.2%, honey 56.0%) and over half receiving antibiotics (CCO 57.4%, honey 55.7%). The most commonly noted comorbidity was hypertension (both 18%) followed by coronary heart disease (CCO 13.7%, honey 7.0%; *p* = 0.003) and diabetes (both 11%). A higher percentage of patients in the honey cohort were prescribed pain medication (CCO 17.0%, honey 22.6%; *p* = 0.052) compared with the CCO cohort (Table 2).

**Table 2.** Patient demographic and clinical characteristics

*Demographic and Clinical Characteristics*	*Clostridial Collagenase*	*Medicinal Honey*	p	*Standardized Difference*^a^
No. of patients	446	341		
Age, mean (SD)	66.2 (20.3)	63.6 (20.5)	0.072	N/A
Age group			0.212	
18–40	14.1%	17.0%		7.96
41–64	26.9%	30.2%		7.31
65+	59.0%	52.8%		12.48
Sex			0.469	
Female	47.8%	45.2%		
Male	52.2%	54.8%		
Ethnicity			0.027	
Caucasian	66.4%	73.0%		
African American	12.1%	9.4%		
Hispanic	12.3%	6.7%		
Other/Unknown	9.2%	10.9%		

^a^ Used in propensity score matching.

N/A, not applicable; SD, standard deviation.

Wound assessment at first visit (Table 3) found that the majority of PUs in the matched cohorts had 0% granulation (CCO 77.2%, honey 77.0%) with >4 concurrent wounds (CCO 56.7%, honey 51.6%). Wound age was most commonly <28 days (CCO 46.8%, honey 43.7%), with mean (SD) wound depth of 0.5 (0.8) cm, and minimal exudate (CCO 42.7%, honey 46.4%). PUs were predominantly at stage III (CCO 56.1%, honey 55.3%) with an infection surrogate score of 0 (CCO 40.0%, honey 36.6%) and found most commonly in the sacrum or buttock areas.

**Table 3.** Wound demographic and clinical characteristics

*Wound Characteristics*	*Clostridial Collagenase*	*Medicinal Honey*	p	*Standardized Difference*^a^
Total wounds	517	517		
Granulation % at first visit, mean (SD)			0.941	
0%	77.2%	77.0%		
0.1–50%	11.6%	9.5%		
>50%	11.3%	13.5%		
No. of concurrent wounds			0.289	
1	18.0%	18.2%		0.50
2	11.6%	14.9%		9.71
3	13.7%	15.3%		4.39
4+	56.7%	51.6%		10.11
Wound age (days) at first visit			0.787	
0–28	46.8%	43.7%		6.22
28.1–75	22.4%	23.8%		3.21
75.1–182.5	12.8%	13.9%		3.41
>182.5	18.0%	18.6%		1.50
Wound surface area (sqcm) at first visit			0.777	
≤0.5	21.9%	20.3%		3.79
0.6–2.0	21.7%	22.1%		0.94
2.1–10	32.1%	31.9%		0.41
>10	18.2%	20.7%		6.36
Missing	6.2%	5.0%		5.05
Wound depth (cm) at first visit^b^, mean (SD)	0.5 (0.8)	0.5 (0.8)	0.767	
PU stage			0.989	
Unstageable	3.3%	3.1%		1.10
II	13.5%	13.9%		1.12
III	56.1%	55.3%		1.56
IV	27.1%	27.7%		1.30
PU location			0.086	
Sacrum/buttock	32.3%	36.6%		
Other site	25.7%	28.6%		
Heel	19.7%	15.7%		
Hip/thigh	8.5%	6.8%		
Ankle	7.5%	5.0%		
Back	6.2%	7.4%		
Wound exudate level at first visit			0.048	
None	27.1%	20.5%		
Minimal	42.7%	46.4%		
Moderate	26.3%	27.1%		
Heavy	3.9%	6.0%		

^a^ Used in propensity score matching.

^b^ Missing results for 12% of clostridial collagenase and 16.8% of medicinal honey cohorts

sqcm, square centimeter.

Honey-treated PUs underwent significantly more selective sharp debridements, with 51.5% receiving a total of three or more compared with 29.6% of CCO PUs (*p* < 0.001) and similarly higher mean (SD) numbers of selective sharp debridements (CCO 2.7 [5.2], honey 4.4 [5.8]; *p* < 0.001), as well as wounds receiving negative pressure wound therapy (CCO 29.0%, honey 38.3%; *p* = 0.002) (Table 4).

**Table 4.** Treatment characteristics

*Treatment Characteristic*	*Clostridial Collagenase*	*Medicinal Honey*	p
All wounds, *n* (%)	517 (100)	517 (100)	
Days to first application, mean (SD)	18.1 (65.3)	53.6 (143.2)	<0.001
Days of use, mean (SD)	34.0 (54.2)	33.6 (60.2)	0.904
Total visits, mean (SD)	9.1 (9.9)	12.6 (16.6)	<0.001
Treated visits, mean (SD)	3.3 (3.6)	3.1 (2.8)	0.201
% Treated visits, mean (SD)	50.6 (31.5)	41.8 (31.1)	0.085
No. of debridements–total			<0.001
0.0	39.7%	21.7%	
1.0	18.8%	13.7%	
2.0	12.0%	13.3%	
3.0–4.0	12.2%	19.9%	
≥5.0	17.4%	31.3%	
No. of debridements per 4 weeks			<0.001
<2.0	80.7%	67.3%	
2.0–3.9	14.5%	23.6%	
≥4.0	4.8%	9.1%	
Concurrent therapies			0.042
Negative pressure wound therapy	29.0%	38.3%	
Hyperbaric oxygen therapy	2.1%	1.2%	
Becaplermin gel or cellular tissue products	1.4%	4.4%	

Patients were treated (days of use) for a mean (SD) of 34.0 (54.2) days and 33.6 (60.2) days, respectively (*p* = 0.904). Significantly fewer mean (SD) total visits were required by CCO-treated PUs (CCO 9.1 [9.9] visits, honey 12.6 [16.6] visits; *p* < 0.001), with treatment administered at an average (SD) of 50.6% (31.5%) and 41.8% (31.1%) of visits, respectively (*p* = 0.085). These observations remained relatively consistent for each of the subsets of patients who achieved 100% granulation at 1 year and those with epithelialization at 1 year.

### Granulation results at 1 year

A significantly greater percentage of CCO-treated PUs achieved 100% granulation at 1 year (CCO 42.0%, honey 35.2%; *p* = 0.025) (Table 5). PUs treated with CCO were 38% more likely to achieve 100% granulation at 1 year compared to honey-treated PUs based on logistic regression modeling (OR 1.384, 95% confidence limit [CL] 1.057–1.812, *p* = 0.018) (Table 6).

**Table 5.** Observed granulation and epithelialization at 1 year

*Outcomes*	*Clostridial Collagenase* n = 517	*Medicinal Honey* n = 517	p
100% Granulation at 1 year, *n* (%)	217 (42.0)	182 (35.2)	0.025
Weeks to 100% granulation at 1 year, mean (SD)	36.5 (20.0)	40.3 (18.2)	0.001
Days to 100% granulation at 1 year, mean (SD)	255.3 (129.8)	282.2 (127.4)	<0.001
Epithelialization at 1 year, *n* (%)	146 (28.2)	110 (21.3)	0.009
Weeks to epithelialization at 1 year, mean (SD)	41.2 (18.4)	44.0 (16.7)	0.011
Days to epithelialization at 1 year, mean (SD)	288.6 (128.9)	308.1 (116.6)	0.011

**Table 6.** Logistic regression for 100% granulation at 1 year

*PU Treatment Characteristic*	*OR*	*Lower 95% CI*	*Upper 95% CI*	p
Clostridial collagenase (vs. honey)	1.384	1.057	1.812	0.018
Patient has home healthcare	1.934	1.463	2.557	<0.001
Wound age >182.5 days^a^	0.656	0.438	0.983	0.041
Infection surrogate score 3–6^a^	0.623	0.416	0.932	0.021
Infection surrogate score 2^a^	0.517	0.325	0.824	0.005
2.0–3.9 debridements/4 weeks^a^	0.609	0.414	0.896	0.012
≥4.0 debridements/4 weeks^a^	0.265	0.125	0.563	0.001
PU location—heel^a^	0.567	0.374	0.860	0.008
Patient age 80+ years^a^	0.505	0.323	0.790	0.003

Covariates shown are those significantly (*p* ≤ 0.05) affecting the odds of 100% granulation.

^a^ Reference values: wound age 0–28 days, infection surrogate score 0, debridements/4 weeks <2.0, PU location buttock, patient age 18–40 years.

CI, confidence interval; OR, odds ratio.

**Figure 1.**
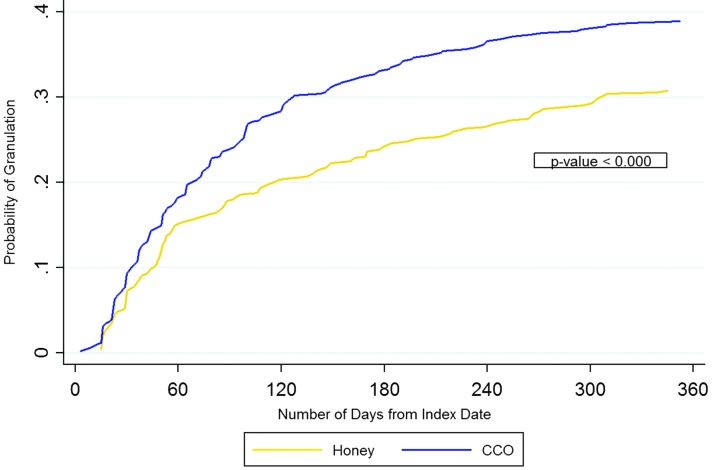
Probability of 100% granulation over 365 days (Kaplan–Meier curve).

The Cox regression found that CCO-treated PUs had a 30% significantly higher probability of achieving 100% granulation compared to honey-treated PUs, with a HR of 1.302 (95% CL 1.056–1.605, *p* = 0.013). CCO-treated PUs achieved 100% granulation within a significantly shorter mean (SD) time frame compared to honey (CCO 255.3 [129.8] days, honey 282.2 [127.4] days; *p* < 0.001) (Table 5). Figure 1 graphically shows the probability of achieving 100% granulation during the 1-year follow-up, comparing CCO and honey cohorts.

### Epithelialization results at 1 year

Logistic regression modeling of epithelialization at 1 year (Table 7) found that CCO-treated PUs were 47% more likely to epithelialize compared to honey-treated PUs (OR 1.467, 95% CL 1.051–2.047, *p* = 0.024). Significantly higher proportions of PUs treated with CCO achieved epithelialization at 1 year (28.2% vs. 21.3%, *p* = 0.009) (Table 5).

**Table 7.** Logistic regression for epithelialization at 1 year

*PU Treatment Characteristic*	*OR*	*Lower 95% CL*	*Upper 95% CL*	p
Clostridial collagenase (vs. honey)	1.467	1.051	2.047	0.024
2.0–3.9 visits/4 weeks^a^	1.547	1.025	2.335	0.038
Patient has home healthcare	1.422	1.005	2.014	0.047
Male	0.689	0.488	0.972	0.034
Wound depth 0.1–0.3 cm^a^	0.620	0.407	0.946	0.027
Wound depth ≥0.3 cm^a^	0.559	0.339	0.921	0.022
Wound age >182.5 days	0.530	0.308	0.911	0.022
Infection surrogate score 2^a^	0.524	0.303	0.937	0.029
Infection surrogate score >6^a^	0.454	0.237	0.869	0.017
Age 80+ years^a^	0.493	0.270	0.899	0.021
Wound surface area >10.0 sqcm^a^	0.471	0.258	0.861	0.014
Ethnicity—other^a^	0.446	0.230	0.866	0.017
≥4 debridements/4 weeks	0.417	0.176	0.988	0.047
Patient has paralysis	0.375	0.176	0.800	0.011
Arrival score 10^a^	0.288	0.092	0.896	0.032

Covariates shown are those significantly (*p* ≤ 0.05) affecting the odds of 100% granulation.

^a^ Reference values: visits/4 weeks <2, wound depth ≤0.1 cm, wound age 0–28 days, infection surrogate score 0, patient age 18–40 years, wound surface area ≤0.5 sqcm, ethnicity white, debridements/4 weeks <2.0, and arrival score 0.

**Figure 2.**
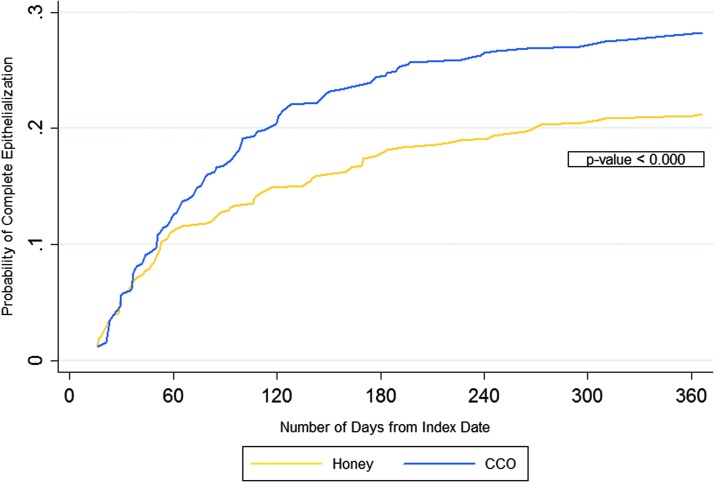
Probability of complete epithelialization over 365 days (Kaplan–Meier curve).

The Cox regressions found that CCO-treated PUs had a 42% higher probability of epithelialization at 1 year (HR 1.421, 95% CL 1.0909–1.8516, *p* = 0.009). Figure 2 contrasts the probability of CCO and honey PUs achieving epithelialization during the 1-year follow-up period. The mean (SD) number of days for achieving epithelialization was significantly lower for CCO-treated PUs treated with CCO than those treated with honey at 1 year (288.6 [128.9] days vs. 308.1 [116.6] days, *p* = 0.011; Table 5).

## Discussion

This comparative analysis of enzymatic debridement with CCO and autolytic debridement with support from medicinal honey found for the primary outcomes of granulation and epithelialization that CCO-treated wounds were 38% more likely to achieve 100% granulation at 1 year and in a significantly shorter time frame (255 vs. 282 days) and 47% more likely at 1 year to achieve epithelialization also in shorter time frames (289 vs. 308 days) compared to matched PUs treated with honey. Healthcare resource use was also lower for CCO-treated PUs compared to honey with significantly fewer total visits (9.1 vs. 12.6), fewer total selective sharp debridements (2.7 vs. 4.4), and fewer PUs receiving negative pressure wound therapy (29% vs. 38%). These findings of more complete and faster granulation and epithelialization with CCO (vs. honey) bear important implications from a clinical perspective, as well as impacts to the economic burden of this costly adverse health condition.

Medical complications that can develop from chronic wound PUs can wreak havoc on patients' health and quality of life. Mechanisms to support faster and more complete PU healing may prevent these sequelae from occurring sooner, providing more days of relief for the patient and their care team.

PUs can impose on the patient, their caregivers, and healthcare professionals a costly cycle of hospitalizations, clinic visits, and home healthcare requiring extensive management resources from a trained multidisciplinary team. In their 2015 study, Woo *et al.* found the cost of debridement dependent on the resources required over the length of time needed to achieve a clean wound bed, with surgical sharp and enzymatic debridement methods requiring the shortest durations and fewest clinical visits and, thereby, the two most cost-effective debridement methods. Autolytic debridement was said to take the longest to work, relying on the patient's own cellular mechanisms to remove necrotic tissue.^17^

There has been a paucity of comparative studies with medicinal honey, and this study is the first we are aware of comparing enzymatic debridement to autolytic debridement with medicinal honey. A number of studies have compared enzymatic debridement with CCO to other debridement methods, primarily other autolytic modalities, in terms of both treatment efficacy and cost. A US-based, prospective, randomized, parallel-group, open-label exploratory study of diabetic foot ulcers with 55 participants receiving CCO plus serial sharp debridement compared with supportive care plus serial sharp debridement found that CCO-treated ulcers healed more quickly and completely while costing ∼$300 less.^40^ An economic analysis based on a RCT of PU therapy in a long-term care setting found CCO more cost-effective relative to autolysis with hydrogel dressings.^41^ Muller *et al.* in an RCT of patients, with grade IV heel pressure sores, found that 91.7% of the collagenase patients were treated successfully compared with 63.6% of the patients in the hydrocolloid group (*p* < 0.005) with more rapid closure, further showing collagenase treatment to be more cost-effective than the hydrocolloid treatment owing to the shorter closure time.^42^ An RCT of long-term care facility PU patients comparing CCO to autolysis demonstrated that at 42 days of therapy 85% of the CCO-treated patients achieved a clean wound bed without initial or concomitant sharp debridement compared with 29% in the autolysis arm.^43^ In a 1999 study by Mosher of elderly long-term care residents, CCO compared to autolysis, wet-to-dry dressings (mechanical debridement), or fibrinolysin debridement had the highest probability of achieving a clean wound bed, low infection rate, and lowest total cost.^44^ A recent observational, retrospective study compared treatment of stage IV PUs using enzymatic debridement plus selective debridement with selective debridement alone, finding the CCO treatment arm significantly more likely to achieve greater and faster epithelialization 1 and 2 years after treatment initiation with greater wound closure at 1 and 2 years (22% vs. 11% after 1 year and 27% vs. 14% after 2 years, respectively).^32^ Our results in terms of achieving 100% granulation and epithelialization at 1 year were similar in both the proportion of patients and the time to closure for CCO in these studies and for honey as an autolytic support method.

This analysis examined outcomes of using enzymatic debridement and autolytic debridement support with medicinal honey. The choice of debridement using any of the available methods depends on the patient's medical condition and the condition of each wound. For each patient, these are unique. The clinician's decision requires consideration of the patient's goals and condition, the wound characteristics, the care setting and the talents of the other care team members, and importantly, evidence of the efficacy and limitations of the various modalities available.

### Limitations

The USWR EHR data used in this study were collected from over 100 contributing hospital outpatient wound care clinics' billing records to support reimbursement and were not collected for research purposes. There may be variability in the data reported among the various contributing clinics. Diagnoses or procedures may be subject to coding error, for which the extent of miscoding or undercoding that could result in bias is unknown, and may also result in measurement error in ICD-9-CM or CPT based variables. Analyses using text fields instead of discrete numeric data may be subject to error resulting from incorrect interpretation of the data. Patient medical history was limited to the patients' visits to a specific provider and clinic, such that patients' outpatient treatment for other comorbidities and by other clinics (*e.g.,* non-USWR clinics) was unknown. Patients with a qualifying PU diagnosis before the study period may have been incorrectly categorized as incident. Multivariate analyses of outcomes provided further adjustment for potentially confounding covariates used in the matching process and an additional degree of robustness in cohort comparisons; however, there is always the potential for unmeasured confounders, especially since information such as comorbidity severity and other sociodemographic variables (*e.g.,* socioeconomic status, basal metabolic index, smoking status, and so on) were unavailable. The USWR EHR is a convenience sample of contributing clinics treating PUs in the US and so findings in this study may not be generalizable to other types of chronic wounds or to patient populations outside of the wound care clinics contributing data to the USWR EHR.

## Innovation (conclusions)

This is the first retrospective observational study comparing the clinical impact of enzymatic debridement using CCO with autolytic debridement support using medicinal honey for PU management. It found CCO-treated PUs significantly more likely to achieve 100% granulation and epithelialization at 1 year and at significantly faster rates compared to honey-treated PUs, with fewer healthcare visits, less debridement, and less use of negative pressure wound therapy. Enzymatic debridement with CCO provided statistically significant greater clinical benefit than honey for treating PUs at all PU stage levels. Future research evaluating cost-effectiveness of CCO compared to honey in this population is warranted.

Key Findings This observational study examined the clinical impact of enzymatic debridement with collagenase ointment relative to autolytic debridement with medicinal honey for management of PUs.

• Enzymatic debridement with collagenase ointment provided more complete granulation of PUs at 1 year compared with autolytic debridement using medicinal honey.
• Epithelialization at 1 year was more complete and occurred faster for patients using collagenase ointment than those using medicinal honey.
• Enzymatic debridement of PUs with collagenase ointment offered clinically significant advantages over medicinal honey in all stages of PUs.

## About the Authors

**Adrienne M. Gilligan, PhD,** is a Researcher at Truven Health Analytics. She is also an adjunct assistant professor at the University of North Texas Health Sciences Center College of Pharmacy. **Richard Bizier, BS,** is a Senior Programmer Analyst at Truven Health Analytics. **Bong-Chul Chu, PhD,** is a Senior Biostatistician at Truven Health Analytics. **Marissa J. Carter, PhD, MA,** is the Chief Executive Officer for Strategic Solutions, Inc. **Caroline E. Fife, MD,** is the Chief Medical Officer at Intellicure, Inc. and the Medical Director, Wound Clinic at St. Luke's Hospital. **Curtis R. Waycaster, RPh, PhD,** is the Director of Health Economics and Outcomes Research at Smith & Nephew. He is also an adjunct assistant professor at the University of North Texas Health Sciences Center College of Pharmacy.

## Abbreviations and Acronyms

AHRQ: Agency for Healthcare Research and Quality
CCO: clostridial collagenase ointment
CL: confidence limit
CPT: current procedural terminology
EHR: electronic health record
ESRD: end-stage renal disease
HIPAA: Health Insurance Portability and Accountability Act
HR: hazard ratio
ICD-9-CM: International Classification of Diseases, Ninth Revision, Clinical Modification
OR: odds ratio
PU: pressure ulcer
RCT: randomized clinical trial
SD: standard deviation
sqcm: square centimeter
USWR: U.S. Wound Registry
